# Quantitative Tomographic Laser Absorption Imaging of Atomic Potassium during Combustion of Potassium Chloride Salt and Biomass

**DOI:** 10.1021/acs.analchem.2c03890

**Published:** 2022-12-30

**Authors:** Emil Thorin, Eduardo M. Paiva, Florian M. Schmidt

**Affiliations:** Thermochemical Energy Conversion Laboratory, Department of Applied Physics and Electronics, Umeå University, SE-90187Umeå, Sweden

## Abstract

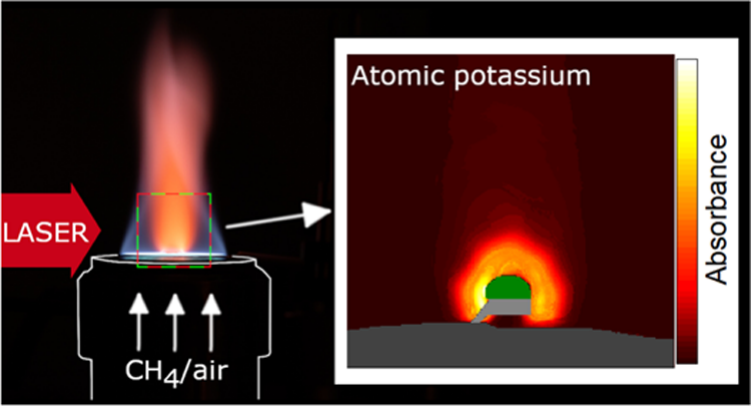

Gaseous potassium (K) species play an important role in biomass combustion processes, and imaging techniques are powerful tools to investigate the related gas-phase chemistry. Here, laser absorption imaging of gaseous atomic K in flames is implemented using tunable diode laser absorption spectroscopy at 769.9 nm and a high-speed complementary metal oxide semiconductor (CMOS) camera recording at 30 kfps. Atomic K absorption spectra are acquired for each camera pixel in a field of view of 28 × 28 mm at a rate of 100 Hz. The technique is used to determine the spatial distribution of atomic K concentration during the conversion of potassium chloride (KCl) salt and wheat straw particles in a laminar premixed CH_4_/air flame with an image pixel resolution of up to 120 μm. Due to axisymmetry in setup geometry and, consequently, atomic K distributions, the radial atomic K concentration fields could be reconstructed by one-dimensional tomography. For the KCl sample, the K concentration field was in excellent agreement with previous point measurements. In the case of wheat straw, atomic K concentrations of around 3 ppm were observed in a cylindrical flame during devolatilization. In the char conversion phase, a spherical layer of atomic K, with concentrations reaching 25 ppm, was found within 5 mm of the particle surface, while the concentration rapidly decreased to sub-ppm levels along the vertical axis. In both cases, a thin (∼1 mm) layer without any atomic K was observed in close vicinity to the particle, suggesting that the potassium was initially not released in its atomic form.

Laser diagnostic techniques have been employed in combustion research for decades for in situ detection of combustion products and trace species.^1−3^ One of the most common methods is tunable diode laser absorption spectroscopy (TDLAS) as it enables fast and calibration-free quantification with robust setups. For many practical applications, line-of-sight (LOS) averaged TDLAS measurements provide sufficient information.^1^ However, thermochemical processes often exhibit both temporal and spatial variations that need to be resolved simultaneously to obtain a better understanding of the process. Chemical imaging based on fluorescence and scattering is frequently employed to address this issue.^3^ Absorption imaging can complement these techniques by providing accurate species quantification.

Early implementations of laser absorption imaging (LAI) with TDLAS typically involved splitting a laser beam into multiple beams to measure the absorption of a target species along a grid of several absorption paths simultaneously, forming a two-dimensional (2D) LOS projection of the species absorption. To resolve the concentrations along the LOS, laser absorption tomography (LAT) can then be applied, which reconstructs the three-dimensional (3D) distribution of the analyte concentration from several 2D projections at different angles using inversion algorithms.^4^ This technique has been applied to imaging of water vapor (H_2_O) and temperature at the exhaust of a jet engine,^5^ in coal-fired combustors,^6,7^ and for hydrocarbon imaging in the cylinders of an internal combustion engine.^8^ Given the large bandwidth of available photodetectors, the advantage of the grid-type implementation of LAT is speed. A major disadvantage is the requirement of a large number of laser beams with accompanying photodetectors to form the measurement grid, resulting in expensive and complex experimental setups. In addition, the spatial resolution is limited by the separation and dimensions of the laser beams.

Employing cameras instead of photodetectors provides higher spatial resolution (μm range) and reduces the complexity of the experimental setup. High-speed cameras today offer frame rates in the kHz to MHz range in the visible to mid-infrared (mid-IR) spectral region, which is sufficiently fast to record pixel-by-pixel absorption spectra for typical TDLAS wavelength scan rates of tens to hundreds of Hz. Recently, LAI and LAT have been implemented in the mid-IR for the quantitative detection of several combustion species and temperature in laboratory flames.^9−11^ Due to the axisymmetric geometry in those experiments, i.e., a cylindrical flame, the radial distributions of concentration and temperature could be reconstructed from a single projection direction, a special case of tomographic reconstruction termed one-dimensional (1D) tomography.^4,12−14^

In the ongoing transformation toward a renewable and more sustainable energy system, biomass plays an important role, not least for heat and power production via combustion. Biomass, however, especially forestry, agricultural, and municipal rest products, is a complex fuel with numerous organic and inorganic trace species that may be released to the gas phase during thermochemical conversion and form compounds harmful for health, environment, and reactors.^15,16^ One of the most hazardous compounds is potassium (K), which may be present as atomic K, potassium hydroxide (KOH), potassium chloride (KCl), or potassium sulfate (K_2_SO_4_). By imaging the spatial distribution of K species close to fuel particles, important information about the K release behavior and chemistry can be obtained.

Atomic K imaging has previously been performed using spontaneous emission and laser-induced fluorescence.^17,18^ Imaging of KOH/KCl and K_2_SO_4_ aerosols has been achieved by photofragmentation fluorescence spectroscopy^19^ and Mie scattering,^20^ respectively. One drawback of the techniques mentioned above is the need for calibration procedures to obtain species concentrations, which is important for, e.g., the validation of numerical particle models.^21^

In this paper, we present quantitative tomographic laser absorption imaging of gaseous atomic K in a large physical domain in the vicinity of KCl salt and biomass particles combusted in a methane/air (CH_4_/air) flat flame. The beam of a tunable diode laser at 769.9 nm is expanded to illuminate the potassium plume in the flame and then imaged onto the sensor of a high-speed complementary metal oxide semiconductor (CMOS) camera. Atomic K absorption spectra are recorded by each pixel at a rate of 100 spectra/s. Utilizing the axisymmetric geometry of the experimental setup, the radial distribution of the atomic K concentration is reconstructed from the absorption images using 1D tomography. The results are compared to previous point measurements. Finally, the technique is, for the first time, applied to quantitative widefield imaging of the atomic K distribution during devolatilization and char burning of a biomass particle converted in the CH_4_/air flame.

## Methods

### Laser Absorption Imaging

For laser radiation of incident intensity *I*_0_ (W/cm^2^) and frequency *ν* (cm^–1^), the transmitted intensity *I*_t_ (W/cm^2^) after passing an absorbing species is given by Beer–Lambert’s law
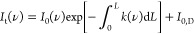
1where *L* is the interaction path length (cm), *k* is the spectral absorption coefficient (cm^–1^), and *I*_0,D_ (W/cm^2^) is the background detector level in the absence of laser radiation. For a single absorption line, the spectral absorption coefficient is given by
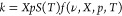
2where *X* is the species concentration (mole fraction), *p* is the pressure (atm), *S* is the absorption line strength (cm^–2^ atm^–1^) at temperature *T* (K), and *f* is the area-normalized line shape function (cm). When using a camera, i.e., an array of detectors, to record the laser intensity, absorption profiles are recorded by each pixel, where each camera frame corresponds to one laser frequency during the TDLAS frequency scan. By integrating eq 2 over all frequencies
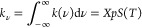
3one obtains the integrated spectral absorption coefficient *k*_ν_ (cm^–2^), from here on referred to as the absorption coefficient. In this way, the absorption profiles recorded by a series of camera frames reduce to a single image of the integrated absorbance. The LOS spectrally integrated absorbance at each pixel *i* is then given by
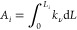
4and can be interpreted as projections of *k*_ν_ along the laser beam line-of-sights with absorption path lengths *L_i_*. The absorption coefficient *k*_ν_ can be deconvoluted using tomographic reconstruction algorithms to obtain the radial concentration distribution, thereby providing additional spatial information of the analyte.

### Tomographic Reconstruction

**Figure 1:**
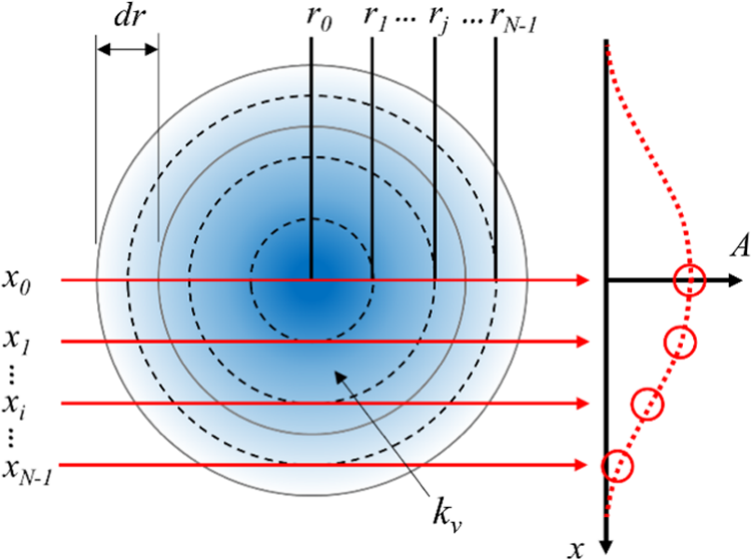
Top view of a horizontal slice of the cylindrical measurement domain. The domain is divided into shells, with radii *r*_*j*_ and thickness d*r*, where uniform conditions are assumed. Each laser beam path interacts with several shells along the LOS, resulting in a projected absorbance *A*_*i*_.

Tomographic reconstruction is a technique to obtain a 3D structure from a series of 2D projections, usually from several directions and angles, and in some special cases, i.e., setups with cylindrical symmetry (as in this work), from a single projection (1D tomography). In the latter case, a horizontal slice can be divided into shells with uniform conditions, also referred to as the “onion peeling” method, illustrated in Figure 1.

A horizontal slice of the measurement domain of radius *R* containing a cylindrically symmetric absorption coefficient can be divided into *N* sections of thickness d*r* = *R*(*N* – 1/2) with radii *r*_*j*_ = *j*d*r*. Light rays at horizontal positions *x*_*i*_ = *i*d*r* interact with different amounts of sections in the measurement volume, and from each ray, projections *A*_*i*_ of the absorption coefficient are obtained. *i*, *j* = 0, 1,···, *N* – 1.

The absorption coefficient at radius *r*_*j*_, denoted *k*_*ν*,*j*_, can be deconvoluted using a series of projections *A*_*i*_, referred to as Abel inversion,^12^ using an inversion matrix^4^
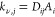
5There are several methods to obtain the inversion matrix *D*_*ij*_, and in this work, the Abel three-point (ATP) method was implemented due to its computational efficiency.^4^ An expression for the inversion matrix using the ATP method was presented by Dasch,^12^ with a typographical correction later published by Villarreal et al.^14^

A drawback of the tomographic reconstruction procedure is that the matrix equation in eq 5 is badly conditioned and that the deconvoluted field variable is sensitive to experimental noise, resulting in oscillatory artifacts in the field variable.^4,13^ The oscillations can be reduced by modifying eq 5 to make the equation less badly conditioned at the cost of solution accuracy. A common method is the Tikhonov regularization, where the field variable is obtained by least-squares fitting the system of equations^22^
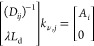
6where *L*_d_ is a discrete derivative matrix and λ is a regularization parameter that controls the degree of regularization. The regularization parameter is chosen to smoothen the solution by suppressing the oscillations while maintaining a similar shape as the unregularized solution. Over-regularization, i.e., choosing a too-high regularization parameter, results in an incorrect shape, while an under-regularized solution does not suppress the oscillations significantly.

### Experimental Setup

**Figure 2:**
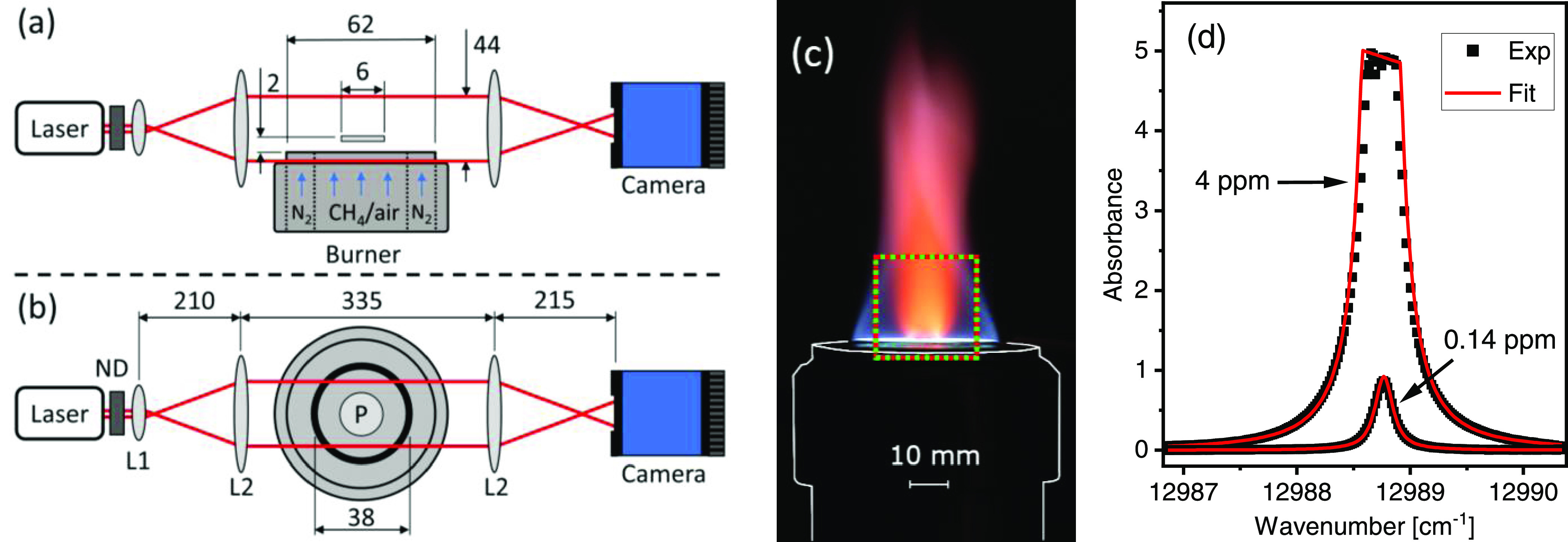
Schematic illustration of the experimental setup, (a) side view and (b) top view. L1 and L2: lenses, ND: neutral density filter, P: Pt plate. Dimensions are given in millimeters. (c) Photograph of the CH_4_/flame and K plume during KCl seeding. The burner is outlined in white, and the imaging domain is indicated by red/green lines. (d) LOS absorption spectra of atomic K (markers) recorded by two different camera pixels. The red, solid lines show Voigt curve fits to the measured data.

The LAI experimental setup is schematically shown in Figure 2a,b. The output beam (diameter 2.2 mm) of a fiber-coupled distributed feedback laser (Nanoplus) emitting at 769.9 nm was expanded to a diameter of 44 mm using a lens-pair (L1 and L2, Thorlabs LB1157-B and LA1353-B). The expanded beam was directed across a cylindrical, water-cooled flat flame burner (Figure 2c) built in-house based on the design by Hartung et al.^23^ The beam center was located 11 mm above the burner surface. Samples of KCl salt or biomass particles were placed on a circular platinum (Pt) plate with 3 mm radius that was located in the center of the burner, suspended on Pt wires 2 mm above the burner surface.

After passing the burner, the expanded laser beam was focused onto a high-speed 12-bit CMOS camera (PCO dimax CS1; 11 μm pixel size), which imaged the laser intensity, as well as the back-illuminated burner and Pt plate. To avoid saturation of the camera sensor, the laser intensity was attenuated before beam expansion using a neutral density filter (Thorlabs NE53B-B). The level of attenuation was chosen to maximize the signal-to-noise ratio without saturating the pixels.

The burner, with a total diameter of 62 mm, had two flow sections with separate gas inlets, a center flow section with a diameter of 38 mm and an outer ring section. A premixed CH_4_/air mixture, controlled by two mass-flow controllers (MKS GM50A), was fed through the center section to produce a stable flat flame with fuel-to-air equivalence ratio (ϕ) of 0.8. The cone-shaped flame was shielded from ambient air by feeding a nitrogen (N_2_) co-flow through the outer section of the burner. The operational burner parameters are summarized in Table 1. Figure 2c shows a photograph of the CH_4_/air flame with the potassium plume emerging in the center due to KCl seeding from the Pt plate.

**Table 1:** Parameters Used to Generate the CH_4_/Air Flat Flame

equivalence ratio	CH_4_[L/min]	air [L/min]	N_2_[L/min]	cooling water [L/min]
ϕ = 0.8	0.775	9.225	5	0.5

The laser wavelength was scanned across the potassium D1 line (769.9 nm) by tuning the laser injection current with a 100 Hz sawtooth wave corresponding to 5.0 cm^–1^ (0.29 nm) peak to peak, generated by one of the outputs of a digital I/O card (National Instruments PXIe-6356). The scan was set to tune the current below lasing threshold to measure the background radiation with the laser off. The second output of the I/O card was used to generate a 30 kHz square wave to trigger the camera exposure, which resulted in 300 frames in a single laser scan. The camera exposure time was set to 12 μs, which provided maximum signal intensity without saturating the camera sensor. The laser beam was imaged on a 240 × 240 pixel grid, which was the maximum number of active pixels for the chosen frame rate and corresponded to a physical domain of 28 × 28 mm. The image pixel resolution was determined using a target of known dimensions, see the Results and Discussion section. Each camera acquisition was set to record 10 laser scans, which, when averaged, resulted in a temporal resolution of 0.1 s for the concentration images.

Figure 2d shows LOS absorption spectra of atomic K (markers) from two different pixels, together with curve fits (lines) using Voigt line shape functions, which revealed LOS-averaged concentrations of 4 and 0.14 ppm. An absorption path length of 20 mm was assumed based on horizontal point measurements of the plume size. The upper spectrum exhibits saturation in absorption around the line center, also known as optically thick conditions, because the low transmitted light intensity in the vicinity of the absorption peak can no longer be resolved by the detector. To obtain the concentration and recover the unsaturated spectrum under optically thick conditions, the curve-fitting procedure reported by Qu et al. was employed.^24^ The high quality of the curve fits, background measurements of the spectra from the CH_4_/air flame, and consultation of spectral databases confirmed the absence of spectral interference from other atomic and molecular species. The presence of soot would cause an overall reduction in light transmission, which would not affect the measured relative K absorption. Distortion of spectra from light extinction by larger particles was not observed.

### Radial Flame Temperature Field

**Figure 3:**
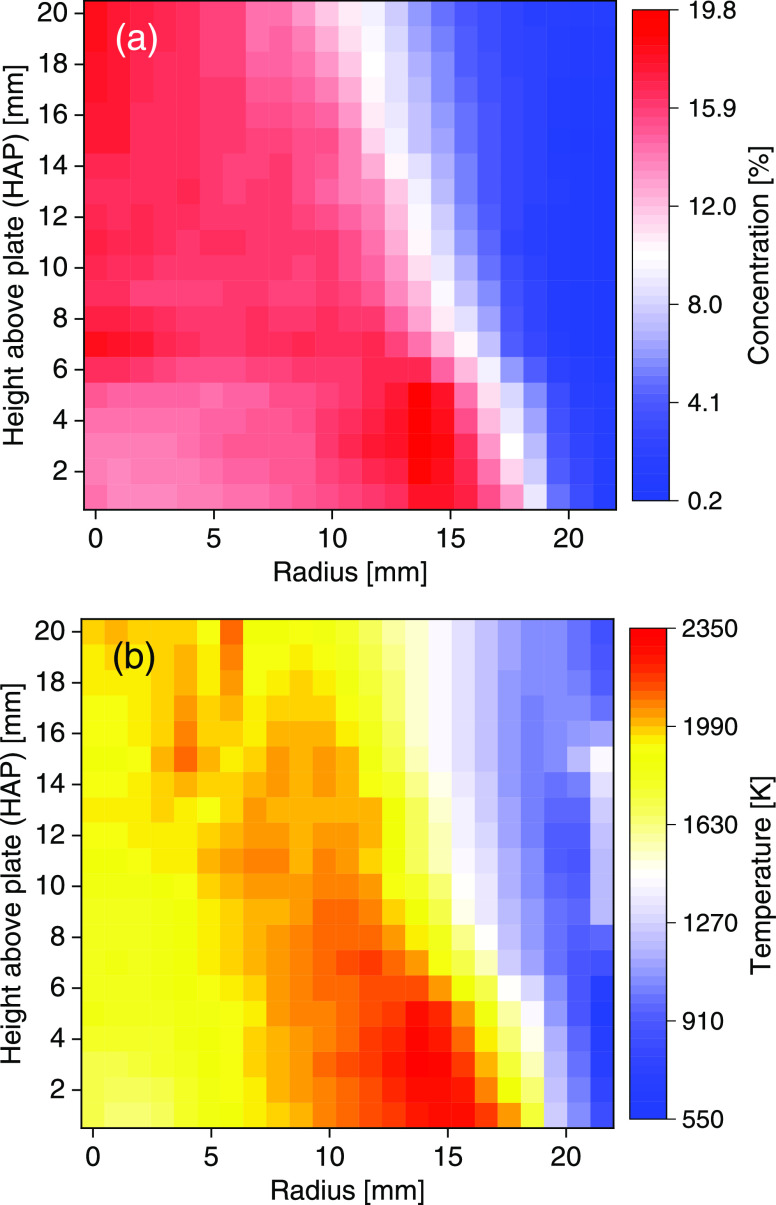
Measured radial (a) H_2_O concentration and (b) temperature fields in the CH_4_/air flame.

Quantification of atomic K in the flame requires knowledge about the temperature distribution in the flame. To this end, a wavelength modulation spectroscopy (WMS) setup for the detection of H_2_O around 1.4 μm was used to measure the flame temperature using two-line thermometry. The system has previously been validated in CH_4_/air flames.^24^ Since 1.4 μm was outside the wavelength range of the CMOS sensor, a series of point measurements were performed. The LOS absorbance of H_2_O was measured as a function of height above the plate (HAP) and horizontal distance from the center of the burner at 20 and 23 positions, respectively. Micrometer translation stages were employed to move the burner. The measured absorbance was deconvoluted and regularized using eq 5 and the procedure described in the Methods section. A regularization parameter of λ = 0.5 provided a sufficient smoothing while also maintaining the shape of the unregularized field. The radially resolved H_2_O concentration and the temperature field determined using two-line thermometry are shown in Figure 3a,b, respectively.

Close to the Pt plate, the CH_4_/air flame was cylindrical and had a radius of 18 mm, in accordance with the size of the center section of the burner. High temperatures are observed at a radius of 15 mm, but above the plate, there is a clear obstruction of the flame leading to a ∼500 K lower temperature. Toward the right edge of the image (20 mm radius), an increase in temperature can be observed at around 12 mm HAP. In this region, the H_2_O concentration is low, which hampers the temperature measurement. Thus, the temperature in this region can be assumed to be overestimated. This does not affect the quantification of atomic K imaging, which is imaged within a radius of 14 mm (image size of ∼28 × 28 mm).

The maximum measured temperature is higher than the adiabatic CH_4_/air flame temperature (∼2000 K for a stabilized flat flame at ϕ = 0.8), probably due to a combined effect of the noise sensitivity of the deconvolution algorithm and the sparse sampling in these measurements. However, this should have a small influence on atomic K concentration as the temperature sensitivity of the line strength is rather low in this temperature range; <5% per 100 K. The radial temperature field was interpolated to the same data binning as the atomic K images to obtain the line strength at each pixel and calculate the atomic K concentration using eq 3.

### Image Processing

The raw data for a single atomic K concentration image consists of a data cube (*x*, *y*, *t*), where *x* and *y* represent the spatial dimensions of individual 240 × 240 pixel images of the laser intensity and *t* represents the time dimension of 3000 consecutive images from 10 laser scans, reduced to 300 after averaging. Diffraction patterns in the form of airy disks were observed in the raw intensity images and resulted in errors in the evaluated concentrations, which showed similar airy-disk patterns. To suppress the diffraction pattern, a computational diffraction removal scheme was applied, similar to what is presented by Schwarm et al.^10^ A two-dimensional Fourier transform was computed for all (*x*, *t*) slices of the data cube to obtain slices in the frequency domain, x* and t* denoting the spatial and temporal frequencies, respectively. A super-Gaussian filter of order *n* and width *w* (*n* = 1: Gaussian function)
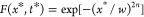
7was applied on all slices and then inversely Fourier transformed, resulting in a filtering of the airy pattern in the *x*-dimension. The filtering was repeated for the *y*-dimension. Using a filter of order 2 and width of 8 px^–1^ was found to be a good compromise of diffraction suppression without distorting the general structure of the images.

After diffraction removal, the images were spatially averaged with a 3 × 3 px average for each pixel. The spectral absorbance profiles were obtained by least-squares fitting a Voigt profile^25^ to the measured absorbance from each pixel, from which the integrated absorbances *A*_*i*_ were calculated. Curve-fitting was necessary, since, under optically thick conditions, the full absorption profile cannot be resolved properly, which would lead to an incorrect integrated absorbance. Using the integrated absorbances, the radial absorption coefficients *k*_*v*,*j*_ can be deconvoluted employing the tomographic inversion procedure presented in the Methods section, and the radial concentrations can be calculated using eq 3.

To summarize, the data evaluation sequence consisted of (1) temporally average intensity images of 10 laser scans, (2) suppress diffraction pattern by Fourier filtering, (3) 3 × 3 px spatial averaging of intensity images, (4) fit Voigt profiles to the measured absorption profiles for each pixel, (5) tomographic reconstruction of absorption coefficient *k*_ν_ with regularization, (6) calculate the radial concentration field with eq 3, using *k*_ν_ and the temperature field.

## Results and Discussion

### Image Pixel Resolution

**Figure 4:**
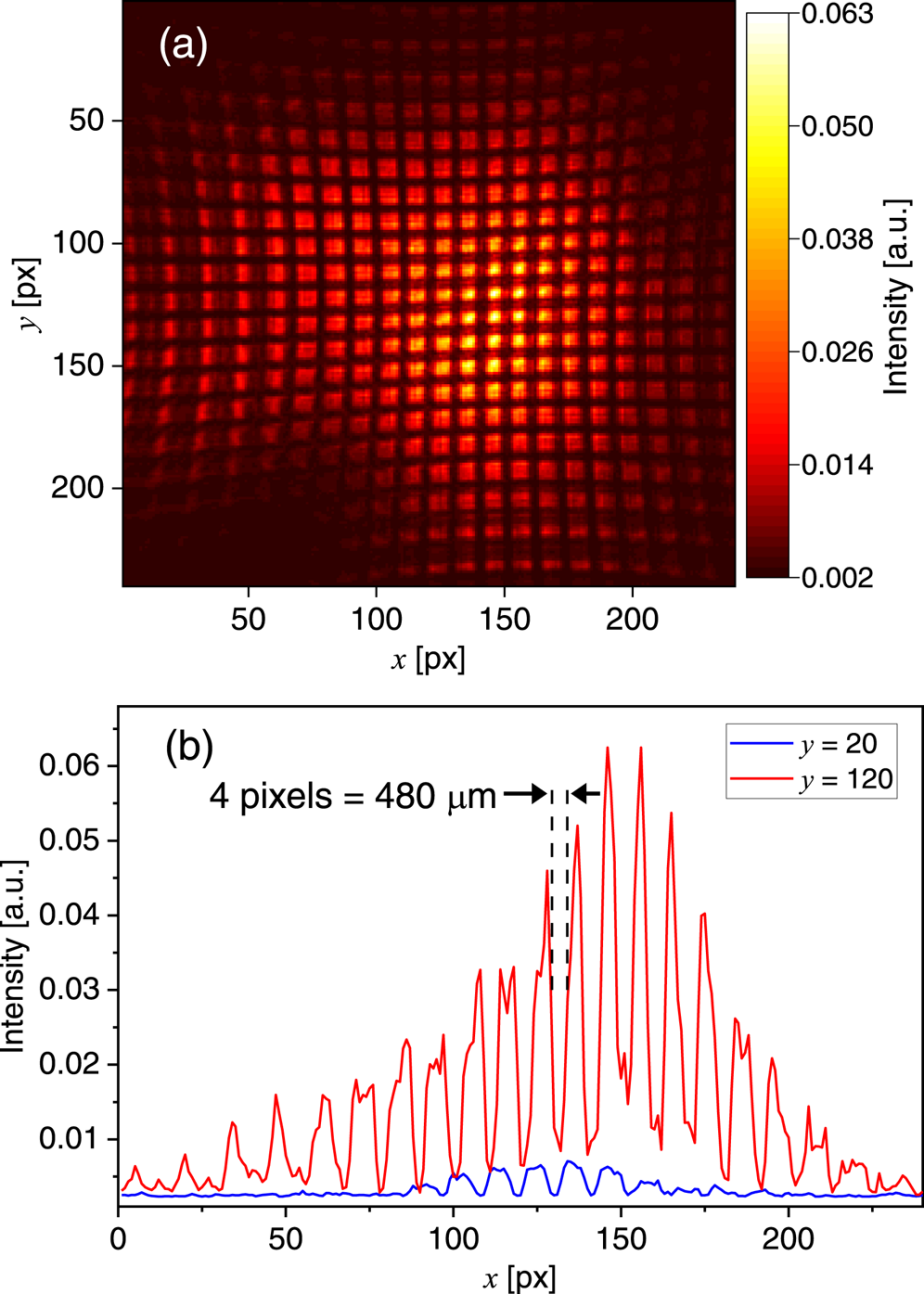
(a) Raw, unfiltered intensity when imaging a back-illuminated wire mesh with 0.5 mm thickness and 1.0 mm separation. (b) Horizontal cross-sections at *y* = 20 (blue) and *y* = 120 (red). The black arrows and dashed lines mark the pixel range used to determine the image pixel resolution.

The pixel resolution in both horizontal (*x*) and vertical (*y*) direction was assessed by imaging the laser intensity when obstructed by a stainless-steel wire mesh with 0.5 mm wire thickness and 1.0 mm spacing between wires. The image of the back-illuminated mesh is shown in Figure 4a. Horizontal and vertical cross-sections were extracted from the images, with two examples of horizontal cross-sections shown in Figure 4b. The pixel resolution was determined from the thickness of the wires divided by the number of pixels that make up the wire shadow, which was taken as half the peak-to-valley intensity indicated by arrows in Figure 4b. This resulted in an image pixel resolution of 120 μm/pixel in both directions (4 pixels on average), which corresponds well to the theoretical image pixel resolution (camera pixel size/magnification) of 117 μm/pixel. The 3 × 3 binning then lead to an effective resolution of 360 μm/pixel for gaseous atomic K. There was no influence of the super-Gaussian filter on the LOS intensity field, and the chosen regularization parameter (λ = 0.5) did not change the smooth atomic K gradients in the radial field.

The image and cross-sections presented in Figure 4 reveal a slight curvature of the object, which is caused by aberrations in the optical system. The center of the image is least affected by aberrations, which is why the center of the image was used for assessment of the pixel resolution. Based on this pixel resolution, the difference in object thickness between the center and the edges is approximately 1 mm in both image directions. Even though the aberrations did not significantly affect the obtained results, aberration-free optics could be used in a future optical system to avoid this artifact. Alternatively, Figure 4a can be used to correct for the aberrations.

It should be noted that for larger objects, such as the biomass particles used in this work, the intensity gradients at the object edges decreased, resulting in an increased uncertainty in the edge position, and thus a lower spatial resolution, down to 2 mm. In the final images, this was accounted for by using masks for the particles that were slightly larger than the actual particle size, covering the full edge uncertainty. In the following, the *x*- and *y*-axes are referred to as “horizontal position” and “height above plate (HAP)”, respectively, and their scale is based on the image pixel resolution stated above.

### KCl Seeding

**Figure 5:**
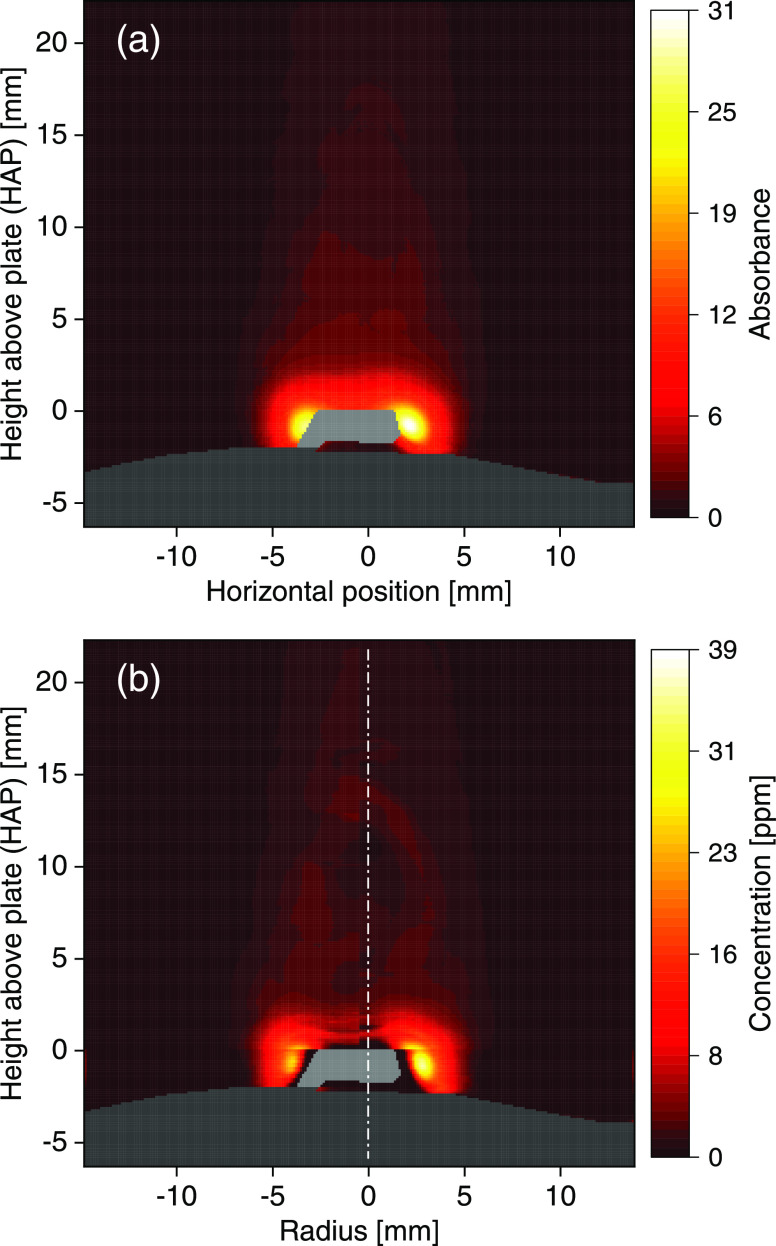
(a) Line-of-sight projection of atomic K absorbance during conversion of a KCl salt sample in the CH_4_/air flame. (b) Two-sided radial reconstruction of atomic K concentration in the flame. The burner surface and Pt plate/wire appear as gray areas. The vertical dash-dotted line (white) denotes the axis of symmetry.

Figure 5a shows a typical image of the LOS-integrated absorbance of atomic K measured during conversion of a KCl sample in the flame. In this figure, the burner, Pt plate, and Pt wire are masked out as gray areas. Given the 3 × 3 pixel spatial average, the effective image pixel resolution was 360 μm. The *x*-axis was centered around the plume at 0.24 mm HAP.

As can be seen in Figure 5a, there are regions of high absorbance around the Pt plate edge, extending even below the plate, and the absorbance rapidly decreases as a function of HAP. The accumulation of atomic K near the plate edge has been observed in previous computational fluid dynamics (CFD) simulations and can be explained by recirculation due to the obstruction of the flow by the plate.^26^ Above approximately 4 mm HAP, the absorbance resembles a Gaussian shape as a function of horizontal position. Moreover, the spatial distribution of the integrated absorbance is symmetric, as required for 1D tomographic reconstruction.

To obtain the radial atomic K concentrations, the atomic K absorbance image was split in half at the center of the measured horizontal absorbance profile as only one-half of the absorbance profile is used in the reconstruction algorithm due to the axisymmetry. The two halves were separately deconvoluted and regularized, and the atomic K concentrations for each half were calculated using eq 3 with the temperature field shown in Figure 3b. Here, a regularization parameter of λ = 0.5 resulted in sufficient smoothing without distorting the image structure. Figure 5b shows the resulting radial atomic K concentration fields from the corresponding halves of the absorbance image (separated by a white dash-dotted line), which are in good agreement with previous CFD simulations.^26^ A discontinuity can be observed at the plate boundary (HAP 0 mm) as the K concentration was set to zero at the plate (boundary condition) during evaluation.

**Figure 6:**
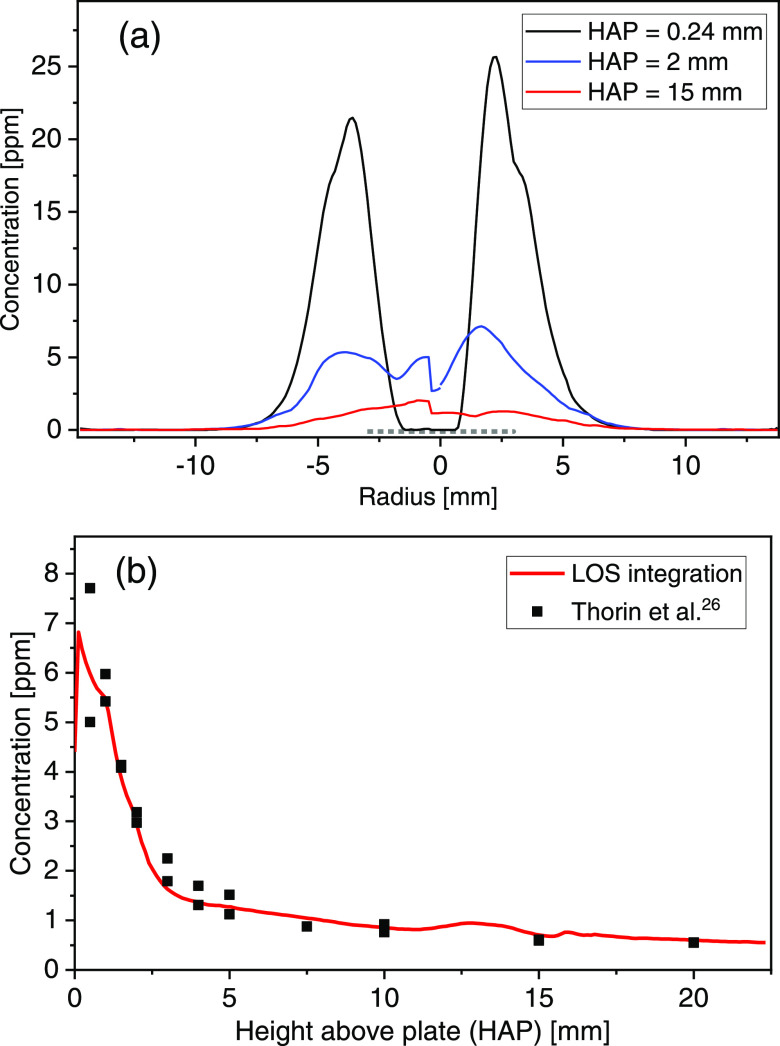
(a) Radial atomic K concentrations above a KCl salt sample at HAPs of 0.24, 2, and 15 mm, extracted from Figure 5b. The Pt plate is indicated by the dotted line. (b) LOS atomic K concentrations at a horizontal position of 0 based on the radial atomic K concentration, as a function of HAP (line) compared to point measurements from previous work (markers).^26^

Figure 6a presents three radial cross-sections extracted from Figure 5b at HAPs of 0.24, 2, and 15 mm, with the Pt plate indicated by a gray dotted line. Close to the plate (<1 mm HAP), the radial distribution shows a high concentration of atomic K around the edge of the plate, while there is practically no atomic K in the center (Figure 6a). The absence of atomic K in the center indicates that the atomic K released from the samples is forced toward the plate edge by the recirculation flow. As the HAP increases, the radial profile transitions toward a Gaussian shape. The cross-sections at 2 mm and 15 mm HAP show sharp variations close to the center. This can be explained by a slight misalignment between the vertical axes of the burner and the camera, which means that the centerline (white dash-dotted line) in Figure 5b should not be vertical, but rather at an angle with respect to the vertical axis of camera. Due to the properties of the inversion algorithm, the error accumulates toward the center.^13^

To compare the imaging results to LOS point measurements of axial atomic K concentrations conducted in a previous study,^26^ the atomic K concentration field was radially averaged at each HAP assuming an absorption path length of 20 mm. Figure 6b shows both the previous point measurements for a KCl sample^26^ and the radially averaged atomic K field measured in the current work as a function of HAP. An excellent agreement is observed, with concentrations approaching thermodynamic equilibrium at about HAP 20 mm. In the previous study, using line-of-sight absorption spectroscopy, the laser beam size posed challenges in evaluating the atomic K concentration close to the plate (<2 mm), but this was not an issue here using LAI and 1D-LAT. The small oscillation in the HAP 11–16 mm region is due to residual diffraction features that could not be removed by filtering during image processing.

### Biomass Conversion

**Figure 7:**
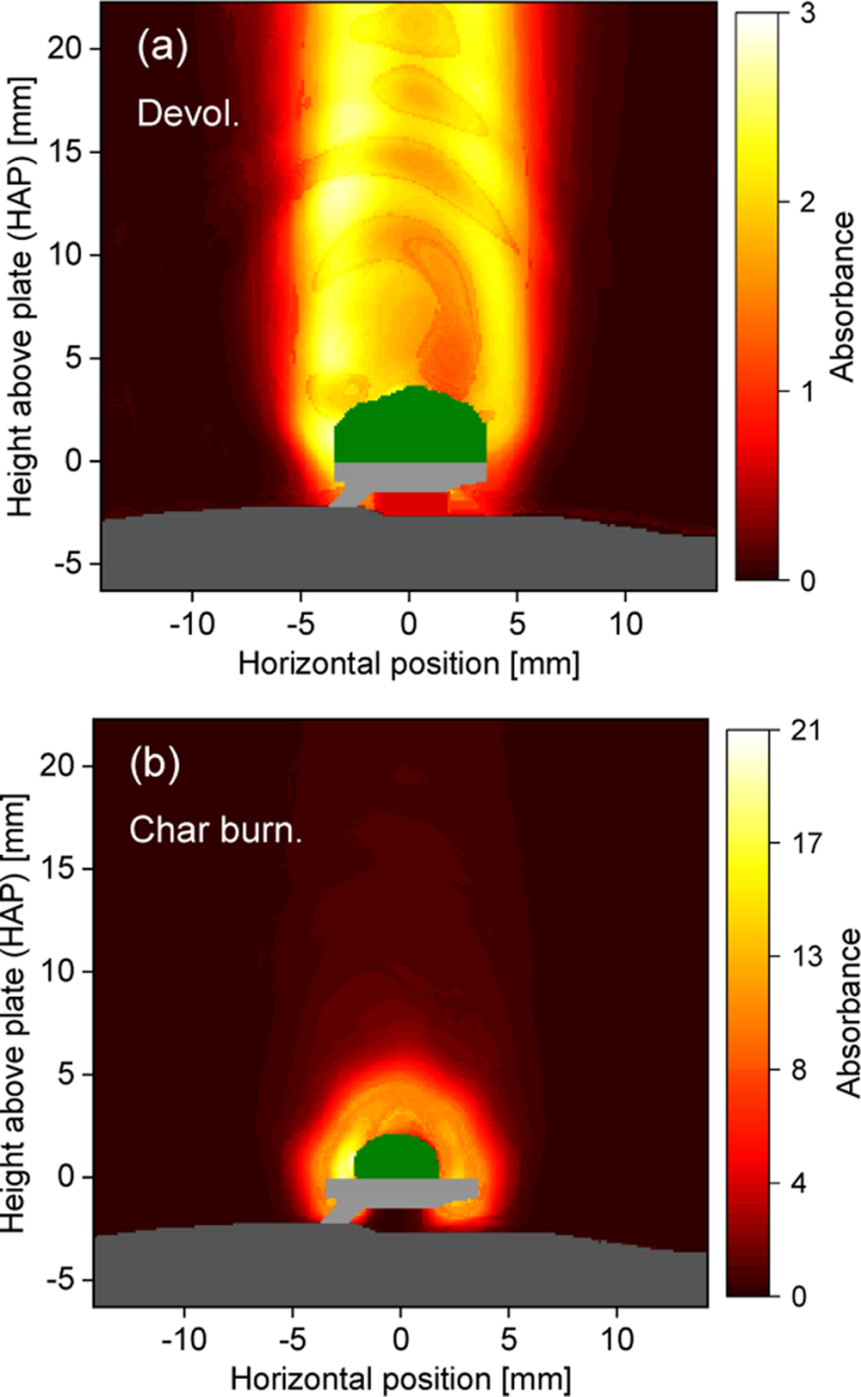
Integrated absorbance of atomic K around a 120 mg wheat straw particle during (a) devolatilization and (b) char burning in the CH_4_/air flame. The biomass particle is shaded in green, while the burner surface and Pt plate/wire appear as gray areas.

A wheat straw pellet was cut and grinded to form a particle with axisymmetric shape and placed on the Pt plate. Figure 7 shows typical images of the integrated absorbance of atomic K in the vicinity of a wheat straw particle (green) weighing 120 mg during (a) the devolatilization stage and (b) the following char burning phase. As for the case with KCl seeding, the symmetric distribution of atomic K absorbance allows for tomographic reconstruction of the atomic K distribution around the biomass particles.

**Figure 8:**
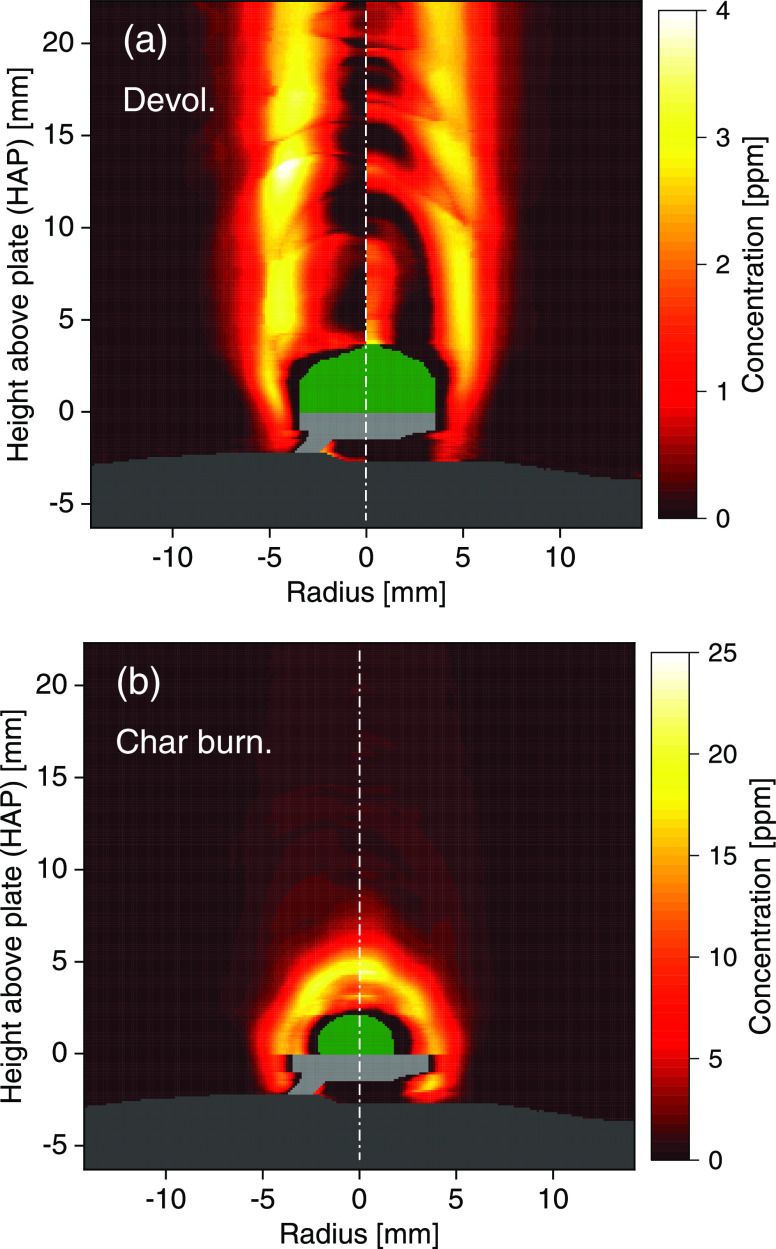
Radial atomic K concentration field around the wheat straw particle during (a) devolatilization and (b) char burning, reconstructed from the data shown in Figure 7. The biomass particle is shaded in green, while the burner surface and Pt plate/wire appear as gray areas. The vertical dash-dotted lines (white) denote the axes of symmetry.

The tomographic reconstructions (λ = 0.5) of the radial atomic K concentrations are presented in Figure 8. Since the temperature field was unknown for those measurements, a fixed temperature of 1800 K was assumed. There is a clear difference in concentration levels between the devolatilization and char burning phases, as observed in previous measurements,^21,26^ and also a clear difference in distribution. In the devolatilization phase, Figure 8a, most of the K atoms can be found in a cylindrical shell with 5 mm radius, and the concentration decreases toward the center. In this phase, the burning particle produces a diffusion flame (biomass flame), where the temperature, and thus the atomic K level, is significantly higher in the shell than in the center region, given the flow obstruction due to the particle and sample holder.^9,27^ The biomass flame extended far (>5 cm) outside the camera field of view, which can explain the rather constant axial concentration within the imaged shell region. These observations suggest that the K atoms were primarily located near the hot reaction zone of the diffusion flame.

**Figure 9:**
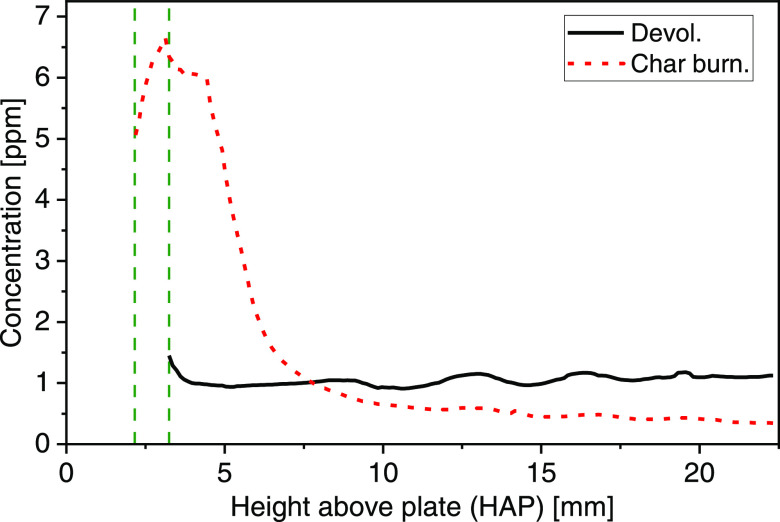
LOS atomic K concentrations above the biomass particle (at a horizontal position of 0) as a function of HAP during devolatilization (solid line) and char burning (short-dashed line), extracted from Figure 8. The vertical dashed lines (green) denote the corresponding particle surfaces.

The concentration oscillations along the vertical direction in the center of the image in Figure 8a are a consequence of the residual diffraction pattern. This could also be the reason for the apparently high concentration at the top boundary of the biomass particle. A high atomic K level there is unlikely, since the concentrations are otherwise low in the vicinity of the particle. The LOS average concentration during devolatilization is extracted from Figure 8a, assuming a path length of 20 mm and shown as a function of HAP in Figure 9. A rather constant atomic K level around 1 ppm was found in the image domain.

During the char burning phase, Figure 8b, there is no longer a biomass flame and the temperature distribution is more homogeneous in comparison to the devolatilization phase. The atomic K is evenly distributed around the particle surface but decreases as a function of distance from the particle, most probably due to the formation of KOH.^21^ The peak concentration level of atomic K is more than five times higher (>20 ppm) than that observed in devolatilization (<4 ppm). This is reasonable as (i) the particle temperature during char conversion is higher, (ii) most of the K in the fuel is released after the devolatilization stage, and (iii) the gas temperature in the vicinity of the particle is closer to the flame temperature.^21,28,29^ Beyond a height above the particle surface of about 5 mm (corresponding to HAP 8 mm), the LOS average concentration decreased to below 1 ppm (Figure 9). This is in good agreement with previous measurements of K species above wheat straw particles in a CH_4_/air flame,^26^ the small differences in absolute values possibly explained by the different particle size used.

The absence of atomic K close to the biomass particles in the radial concentration fields (Figure 8) indicates an initial K release mainly in forms other than atomic K during both devolatilization and char burning. Atomic K is instead formed by decomposition of inorganic K salts and organic char-bound K in the gas phase.^18^ Note that the biomass particles, highlighted in green in Figures 7 and 8, shrink during the conversion process due to drying and loss of volatile matter. In Figure 8b the particle is smaller than in Figure 8a, and this is also why the release starts at different HAPs, as indicated by vertical dashed lines in Figure 9.

Due to the cylindrical flame and concentration gradients in the vicinity of burning solid fuel particles, LOS-averaged measurements obscure the true species concentrations in the vicinity of a particle. The radial concentration fields obtained after 1D tomographic reconstruction give a better picture of the actual concentrations (compare Figures 7 and 8). In addition, contrary to LOS point measurements with TDLAS, LAI can provide snapshots of the concentration fields in a domain comparable to the particle and flame size, i.e., data for a large range of HAPs can be obtained simultaneously. This provides more accurate experimental data for the determination of reaction rates and the development and validation of numerical particle models.

The present LAI setup is fast enough to provide images at above video rate, up to 100 images/s. The time over which concentration field videos can be recorded and saved is limited only by the camera memory. A higher temporal resolution can be achieved by decreasing the field of view (thereby increasing the maximum camera frame rate) or by decreasing the number of spectral sampling points (thereby allowing for a higher laser scan rate). A higher pixel resolution can be realized by increasing the number of active pixels at the cost of frame rate or by adjusting the beam focus on the camera detector at the cost of field of view.

The results presented in this work highlight the importance of imaging also other abundant gas-phase K species, such as KOH and KCl, in addition to atomic K. In the future, this could be achieved by combining LAI and 1D-LAT with photofragmentation absorption spectroscopy^19,26,30^ for simultaneous imaging of KOH and KCl, thereby providing spatially and temporally resolved information of the three major gas-phase K species.

## Conclusions

One-dimensional tomographic laser absorption imaging has been applied to quantitatively image the radial atomic potassium distribution in a 28 × 28 mm physical domain during conversion of KCl salt and biomass in a premixed CH_4_/air flat flame. Images of atomic K concentration fields were recorded with an acquisition rate of 100 Hz and an image pixel resolution of 120 μm in both dimensions, or 0.1 s and 360 μm, respectively, after averaging. The atomic K concentration field during KCl conversion shows an accumulation of atomic K around the edge of the plate caused by flow recirculation and agrees well with previous LOS point measurements and CFD simulations. The radial atomic K concentrations obtained during devolatilization and char burning of wheat straw particles suggest that the K is initially released in other forms than atomic K, such as KCl and KOH, which then partly decompose to form atomic K as intermediate species in the hot reaction zone, before the concentration decreases toward thermodynamic equilibrium. Laser absorption imaging of K species opens up for detailed, quantitative K release studies, which can improve the understanding of the gas-phase K chemistry and release behavior during thermochemical conversion of solid fuels.
